# Association of pre- and post-stroke glycemic status with clinical outcome in spontaneous intracerebral hemorrhage

**DOI:** 10.1038/s41598-019-55610-z

**Published:** 2019-12-13

**Authors:** Kaijiang Kang, Jingjing Lu, Yi Ju, Wenjuan Wang, Yuan Shen, Anxin Wang, Zhentang Cao, Xingquan Zhao

**Affiliations:** 1 Department of Neurology, Beijing Tiantan Hospital, Capital Medical University, China National Clinical Research Center for Neurological Diseases, Center of Stroke, Beijing Institute for Brain Disorders, Beijing, China; 2 China National Clinical Research Center for Neurological Diseases, Beijing, China

**Keywords:** Cerebrovascular disorders, Stroke

## Abstract

In this study, we aimed to disclose the association of pre- and post-stroke glycemic status with clinical outcome in patients with spontaneous intracerebral hemorrhage (sICH). It was a multicenter, prospective, observational cohort study, conducted in 13 hospitals in Beijing from January 2014 to September 2016. The association of admission random blood glucose (RBG), fasting blood glucose (FBG) and hemoglobin A1c (HbA1c) with clinical outcome at 90 days after sICH onset were analyzed comprehensively. Poor outcome was defined as death or modified Rankin Scale (mRS) score >2. The results showed that elevated RBG and FBG were associated with larger hematoma volume, lower GCS, higher NIHSS (*P* < 0.001), and poor outcome, but HbA1c was not (*P* > 0.05). In stratified analysis, the association of poor outcome with elevated FBG or RBG retained statistical significance just in patients without diabetes. Kaplan-Meier curve and Cox regression showed that patients with elevated FBG or RBG had significantly higher risk of death within 90 days (*P* < 0.05). So we conclude that poststroke hyperglycemia was associated with larger hematoma volume, severe neurological damage and poor clinical outcome, but HbA1c was not relevant to hematoma volume or clinical outcome in patients with sICH.

## Introduction

Hyperglycemia has been thought to be related to hematoma expansion, presence and severity of intraventricular extension, perihematomal edema and poor clinical outcome in patients with acute intracerebral hemorrhage (ICH), even in patients without diabetes mellitus (DM)^1–7^. However, early intensive insulin therapy had failed to improve functional outcome after ICH in previous studies^8,9^. Currently, the optimal management of hyperglycemia and the target glucose level in acute ICH remains still unclear^10,11^. It is suggested that poststroke glucose level might not be an ideal prognostic indicator after sICH, and few studies conducted in small cohorts have suggested that hemoglobin A1c (HbA1c), a biomarker of prestroke glycemic status, could serve as a better predictor of poor outcome in patients after sICH^12,13^. However, the association of pre- and post-stroke glycemic levels with clinical outcome has not been fully elucidated in previous studies with small cohorts. In this prospective study with the largest cohort so far, we aimed to disclose the association of pre- and post-stroke glycemic status with initial neurological injury severity and long-term outcome in patients with sICH.

## Materials and Methods

### Study design and population

The study was a multicenter, prospective, observational cohort study, conducted in 13 hospitals in Beijing from January 2014 to September 2016. The study was performed according to the guidelines from the Helsinki Declaration, and was approved by the Institutional Review Board (IRB) of Beijing Tiantan Hospital, Capital Medical University. All patients or their legally authorized representative were given written informed consent according to the requirements established by each site ethics board. Participating centers collected data and submitted it online to the coordinating center of Beijing Tiantan Hospital, Capital Medical University for merging and analysis.

The inclusion criteria include: 1) first-ever acute-onset ICH, 2) age ≥18, and 3) arriving at hospital within 72 hours after onset. The exclusion criteria include: 1) past history of ICH, 2) age <18, 3) arriving at hospital beyond 72 hours after onset and 4) complicated with major comorbidities or late-stage diseases. There were 1964 ICH patients enrolled in our database. In this paper, we also excluded 159 patients with secondary ICH, which attribute to cerebrovascular malformations, aneurysms, anticoagulant therapy, tumor, trauma, cerebral venous thrombosis or hemorrhagic transformation of ischemic stroke. The overview of the included patients in the study and in each subset of analysis were shown in Supplementary material.

### Clinical information

Demographic information, health habits (smoking status, alcohol use), body mass index (BMI), medical history (ischemic stroke, coronary heart disease, hypertension, diabetes mellitus, dyslipidemia), medication history (antihypertensive, antidiabetic, lipid-lowering, anticoagulant and antiplatelet agents), systolic blood pressure (SBP) and diastolic blood pressure (DBP) were collected at admission.

Other diagnosis or complications (hematoma expansion, rebleeding, associated heart disease, infections, gastrointestinal bleeding, deep venous thrombosis) and treatment (antihypertensive, antidiabetic, lipid-lowering, anticoagulant and antiplatelet agents, surgical treatment) during and post hospitalization were also recorded. Surgical treatment included external ventricular drainage, microinvasive hematoma removal, craniotomy evacuation of hematoma and decompressive craniectomy.

### Baseline neurofunctional assessment and neuroradiological review

Baseline neurological deficit was assessed using the Glasgow Coma Scale (GCS) and the National Institutes of Health Stroke Scale (NIHSS). GCS scores were classified as mild disability (>12) or moderate-severe disability (≤12). NIHSS scores were classified as mild-moderate disability (<16) or severe disability (≥16).

We recorded hematoma location (lobar, basal ganglia, thalamus, brainstem, cerebellum), hematoma volume and intraventricular extension according to first computerized tomography (CT) scans, which were completed within 24 hours after admission. Hematoma volume were measured using the ABC/2 method described in previous studies^14,15^, and were classified into large hematoma (≥30 ml) or small hematoma (<30 ml). For patients who arrived at the hospital within 6 hours of onset, CT was re-examined at 24 hours after onset to confirm whether there was a hematoma expansion. Hematoma expansion was defined as an absolute increase in ICH volume greater than 6 ml or an increase of greater than 33% from baseline ICH volume.

### Laboratory examinations

Superficial venous blood was collected for measurement, and random blood glucose (RBG), fasting blood glucose (FBG) and HbA1c were selected for primary analysis. RBG was assessed immediately on admission. Fasting blood glucose was assessed the next morning after admission (at least 8 hours without any food or infusion of sugary liquid). HbA1c was assessed during acute hospitalization within seven days after admission. Since the blood glucose management for ICH was not clearly stated in current guidelines, it was based primarily on the patient’s basic blood glucose levels and the experience of physicians in this study.

Patients were classified into three groups according to their HbA1c, RBG and FBG levels respectively: perfect (HbA1c <6.0%, RBG <7.0 mmol/L, FBG <6.0 mmol/L), fair (HbA1c 6.0–7.9%, RBG 7.0–9.9 mmol/L, FBG 6.0–7.9 mmol/L), and poor (HbA1c≥8.0%, RBG ≥10.0 mmol/L, FBG ≥8.0 mmol/L). The association of HbA1c, RBG and FBG with clinical outcome at 90 days after onset were analyzed comprehensively.

Other laboratory examinations, including white blood cell (WBC) count, platelet (PLT) count, INR, creatinine, total cholesterol (TC), triglycerides (TG), low-density lipoprotein cholesterol (LDL-C), high-density lipoprotein cholesterol (HDL-C), serum homocysteine (Hcy) and high-sensitivity C-reactive protein (HS-CRP) were also collected.

### Patients follow-up and clinical outcome

At 90 days and 1 year after ICH onset, the functional outcomes of all patients were assessed through telephone follow-up interview to obtain information on death and modified Rankin Scale (mRS). We recorded at least two phone numbers of each recruited ICH patients. For patients who were lost to follow-up, we make telephone follow-up interview once a week for at least three times. Telephone follow-up was centralized for all included patients with a standardized interview protocol. The interviewers were trained on the interview protocol.

In this part, we analyzed the association of RBG, FBG and HbA1c levels with 90-day outcome in patients with sICH. The poor outcome was defined as death within 90 days (including death during the acute phase of ICH) or major disability (mRS >2) at 90 days after ICH onset. Of the 1805 patient with baseline data, there were 1515 patients with follow-up data, and other 290 patients were lost to follow up.

### Statistical analysis

The statistical analysis was performed using a commercial statistical software package (SPSS for Windows, version 22.0, IBM-SPSS, Chicago, IL, US). Descriptive statistics include mean and standard deviation for continuous variables, median and interquartile range for ordinal variables, and number and percentage of total for categorical variables. T-test (normal distribution) or Mann-Whitney U test (skewed distribution), chi-square test (categorical variables), and logistic regression were used to analyze the differences of clinical and radiological characteristics between different blood glucose levels. Logistic regression, receiver operating characteristic (ROC) curve, Kaplan-Meier survival method and Cox regression were used to analyze the relationship between different blood glucose levels and clinical outcome. Differences of P < 0.05 were considered statistically significant for two-tailed tests.

## Results

In this study, we consecutively enrolled 1805 sICH patients with ages ranging from 18 to 93 years (mean age was 58 ± 14 years), and 1218 (68%) were male. The frequent prestroke risk factors for cerebrovascular disease included hypertension (n = 1320, 73%), diabetes (n = 272, 15%), dyslipidemia (n = 178, 10%), smoking (n = 572, 36%) and drinking (n = 662, 37%). The median of HbA1c, RBG and FBG were 5.6 (IQR, 5.3–6.2) %, 7.4 (IQR, 6.1–9.3) mmol/L and 5.9 (IQR, 4.9–7.5) mmol/L respectively. The median of baseline GCS, NIHSS and hematoma volume were 14 (IQR, 8–15), 11 (IQR, 4–21) and 15(IQR, 6–32) ml respectively (Table 1). Since the missing values of HbA1c and FBG exceeded 10% of the total number, we compared the baseline characteristics between valid and missing groups of HbA1c and FBG. We found that there were significant differences in GCS, NIHSS, hematoma volume and proportion of IVH extension between the valid and missing groups of HbA1c and FBG. The GCS was relatively lower, while the NIHSS, hematoma volume and the proportion of IVH extension were relatively higher in the missing groups of HbA1c and FBG. In other words, patients in missing group had more severe neurological deficits and may have worse outcomes. Therefore, we cannot rule out the possibility that the association of elevated HbA1c or FBG with initial neurological injury severity and poor outcome may be underestimated. The baseline characteristics of the 1805 patients in different blood glucose levels were shown in Table 2, and the sample size for each subset of analysis were shown in Supplementary material.

**Table 1:** Demographic and clinical characteristics according to valid or missing values of HbA1c, FBG and follow-up.

	Total (n = 1805)	HbA1c, %			FBG, mmol/L			Follow-up		
		Valid (n = 730)	Missing (n = 1075)	*P* value	Valid (n = 1082)	Missing (n = 723)	*P* value	Followed (n = 1515)	Lost (n = 290)	*P* value
Age, mean ± SD	58 ± 14	58 ± 13	58 ± 14	0.567	58 ± 13	57 ± 14	0.114	58 ± 13	56 ± 14	0.015
Male, n (%)	1218 (68%)	519 (71%)	699 (65%)	0.007	748 (69%)	470 (65%)	0.067	1024 (68%)	194 (67%)	0.817
BMI, mean ± SD	25.6 ± 4.2	25.6 ± 4.0	25.5 ± 4.3	0.687	25.6 ± 3.8	25.5 ± 4.6	0.674	25.5 ± 4.2	25.7 ± 3.9	0.588
Systolic BP (mm Hg), mean ± SD	169 ± 29	165 ± 25	172 ± 31	< 0.001	168 ± 28	171 ± 31	0.031	170 ± 29	167 ± 29	0.250
Diastolic BP (mm Hg), mean ± SD	97 ± 17	96 ± 17	98 ± 20	< 0.001	98 ± 18	97 ± 19	0.066	98 ± 19	97 ± 19	0.537
Prior ischemic stroke, n (%)	259 (14%)	113 (16%)	146 (14%)	0.259	156 (14%)	103 (14%)	0.919	231 (15%)	28 (10%)	0.013
Coronary heart disease, n (%)	34 (2%)	14 (2%)	20 (2%)	0.930	19 (2%)	15 (2%)	0.626	32 (2%)	2 (1%)	0.103
History of hypertension, n (%)	1320 (73%)	545 (75%)	775 (73%)	0.391	798 (74%)	522 (73%)	0.837	1112 (74%)	208 (72%)	0.569
History of diabetes mellitus, n (%)	272 (15%)	132 (18%)	140 (13%)	0.003	159 (15%)	113 (16%)	0.587	234 (15%)	38 (13%)	0.307
History of dyslipidemia, n (%)	178 (10%)	83 (11%)	95 (9%)	0.077	102 (9%)	76 (11%)	0.449	152 (10%)	26 (9%)	0.576
Use of anticoagulation, n (%)	18 (1%)	9 (1%)	9 (1%)	0.511	14 (1%)	4 (1%)	0.189	16 (1%)	2 (1%)	0.643
Use of antiplatelet agent, n (%)	267 (15%)	116 (17%)	151 (17%)	0.648	163 (16%)	104 (18%)	0.301	245 (18%)	22 (9%)	0.001
Current smoking, n (%)	572 (36%)	262 (36%)	310 (33%)	0.252	373 (35%)	199 (33%)	0.501	495 (35%)	77 (32%)	0.581
Current drinking, n (%)	662 (37%)	312 (44%)	350 (37%)	0.004	401 (38%)	261 (42%)	0.128	569 (40%)	93 (39%)	0.705
GCS score, median (IQR)	14 (8–15)	14 (12–15)	12 (6–15)	< 0.001	14 (11–15)	12 (6–15)	< 0.001	14 (8–15)	14 (8–15)	0.175
NIHSS score, median (IQR)	11 (4–21)	8 (3–14)	14 (5–25)	< 0.001	10 (3–17)	14 (5–27)	< 0.001	11 (4–21)	11 (4–20)	0.490
Location of hematoma, n (%)				0.002			< 0.001			0.623
Supratentorial lobe	367 (20%)	153 (22%)	214 (26%)		221 (22%)	146 (30%)		324 (25%)	43 (22%)	
Supratentorial deep location	970 (54%)	469 (69%)	501 (61%)		707 (69%)	263 (54%)		837 (64%)	133 (67%)	
Infratentorial	172 (10%)	61 (9%)	111 (13%)		94 (9%)	78 (16%)		150 (11%)	22 (11%)	
Hematoma volume, median (IQR)	15 (6–32)	12 (5–25)	18 (8–40)	< 0.001	15 (6–30)	17 (6–40)	0.017	15 (6–33)	14 (6–30)	0.591
IVH extension, n (%)	584 (32%)	217 (32%)	367 (43%)	< 0.001	361 (36%)	223 (45%)	0.001	517 (39%)	67 (33%)	0.058
Pre-stroke anti-hyperglycemic therapy, n (%)	162 (9%)	90 (13%)	72 (8%)	< 0.001	99 (9%)	63 (10%)	0.661	147 (10%)	15 (6%)	0.034
Post-stroke anti-hyperglycemic therapy, n (%)	296 (16%)	170 (23%)	126 (12%)	< 0.001	205 (19%)	91 (13%)	0.001	258 (17%)	38 (14%)	0.124

HbA1c: hemoglobin A1c; FBG: fasting blood glucose; BMI: body mass index; GCS: Glasgow Coma Scale; NIHSS: National Institutes of Health Stroke Scale.

**Table 2:** Demographic and clinical characteristics according to baseline glycemic levels.

	HbA1c, % (n = 730)				RBG, mmol/L (n = 1651)				FBG, mmol/L (n = 1082)			
	<6.0 (n = 500)	6.0–7.9 (n = 168)	≥8.0 (n = 62)	*P* value	<7.0 (n = 720)	7.0–10.0 (n = 593)	≥10.0 (n = 338)	*P* value	<6.0 (n = 567)	6.0–7.9 (n = 307)	≥8.0 (n = 208)	*P* value
Age, mean ± SD	57 ± 13	62 ± 13	61 ± 11	<0.001	57 ± 14	58 ± 13	59 ± 13	0.178	57 ± 13	61 ± 13	60 ± 13	<0.001
Male, n (%)	361 (72%)	116 (69%)	42 (68%)	0.613	531(74%)	384(65%)	205(61%)	<0.001	409 (72%)	210 (68%)	129 (62%)	0.025
BMI, mean ± SD	25.5 ± 4.2	25.7 ± 3.4	25.8 ± 3.5	0.867	25.4 ± 4.3	25.4 ± 4.1	26.0 ± 4.2	0.135	25.5 ± 4.0	25.8 ± 3.7	25.6 ± 3.4	0.740
Systolic BP (mm Hg), mean ± SD	166 ± 26	164 ± 26	162 ± 20	0.572	165 ± 26	170 ± 30	178 ± 33	<0.001	163 ± 26	172 ± 29	177 ± 29	<0.001
Diastolic BP (mm Hg), mean ± SD	97 ± 17	94 ± 16	94 ± 15	0.142	96 ± 18	98 ± 18	99 ± 19	0.199	96 ± 17	99 ± 18	100 ± 19	0.020
Prior ischemic stroke, n (%)	63 (13%)	39 (23%)	11 (18%)	0.004	94 (13%)	91 (15%)	56 (17%)	0.260	72 (13%)	48 (16%)	36 (17%)	0.209
Coronary heart disease, n (%)	6 (1%)	6 (4%)	2 (3%)	0.112	8 (1%)	12 (2%)	11 (3%)	0.054	5 (1%)	9 (3%)	5 (2%)	0.065
History of hypertension, n (%)	362 (72%)	135 (80%)	48 (77%)	0.106	505 (70%)	435 (74%)	265 (79%)	0.014	402 (71%)	240 (78%)	156 (75%)	0.059
History of diabetes mellitus, n (%)	15 (3%)	69 (41%)	48 (77%)	<0.001	30 (4%)	69 (12%)	145 (43%)	<0.001	24 (4%)	42 (14%)	93 (45%)	<0.001
History of dyslipidemia, n (%)	37 (7%)	33 (20%)	13 (21%)	<0.001	58 (8%)	51 (9%)	50 (15%)	0.001	53 (9%)	23 (8%)	26 (13%)	0.161
Use of anticoagulation, n (%)	4 (1%)	3 (2%)	2 (3%)	0.175	4 (1%)	5 (1%)	4 (1%)	0.480	3 (1%)	5 (2%)	6 (3%)	0.028
Use of antiplatelet agent, n (%)	60 (13%)	42 (28%)	14 (26%)	<0.001	94 (14%)	88 (17%)	65 (23%)	0.006	76 (14%)	55 (19%)	32 (17%)	0.187
Current smoking, n (%)	196 (40%)	44 (27%)	22 (36%)	0.004	267 (39%)	178 (32%)	91 (31%)	0.034	205 (37%)	103 (34%)	65 (33%)	0.202
Current drinking, n (%)	221 (46%)	72 (44%)	19 (31%)	0.104	276 (41%)	239 (44%)	109 (37%)	0.193	231 (42%)	103 (35%)	67 (34%)	0.063
GCS score, median (IQR)	14 (12–15)	15 (12–15)	14 (11–15)	0.437	14 (12–15)	13 (7–15)	9 (5–14)	<0.001	14 (13–15)	14 (9–15)	11 (6–15)	<0.001
NIHSS score, median (IQR)	9 (3–14)	7.5 (2–15)	4.5 (2–13)	0.137	9 (3–15)	13 (5–22)	19 (8–29)	<0.001	8 (2–13)	12 (5–19)	16 (6–25)	<0.001
Location of hematoma, n (%)				0.494				0.001				0.121
Supratentorial lobe	109 (23%)	29 (18%)	15 (26%)		137 (22%)	129 (27%)	70 (27%)		110 (20%)	73 (25%)	38 (20%)	
Supratentorial deep location	317 (68%)	116 (73%)	36 (62%)		434 (69%)	292 (61%)	151 (57%)		387 (72%)	187 (65%)	133 (69%)	
Infratentorial	39 (8%)	15 (9%)	7 (12%)		54 (9%)	60 (13%)	43 (16%)		41 (8%)	30 (10%)	23 (12%)	
Hematoma volume, median (IQR)	15 (6–28)	9 (4–17)	9 (4–20)	<0.001	12 (6–25)	20 (8–41)	20 (6–50)	<0.001	11 (6–25)	19 (8–36)	20 (8–48)	<0.001
IVH extension, n (%)	149 (32%)	47 (31%)	21 (35%)	0.877	163 (26%)	217 (45%)	161 (59%)	<0.001	132 (25%)	128 (43%)	101 (52%)	<0.001
Hematoma expansion, n/N (%)	42/105 (40%)	10/25 (40%)	3/11 (27%)	0.708	44/114 (39%)	36/87 (41%)	10/37 (27%)	0.312	37/98 (38%)	17/44 (39%)	9/22 (41%)	0.962
Surgical treatment, n (%)	80 (16%)	17 (10%)	5 (8%)	0.061	81 (11%)	136 (23%)	77 (23%)	<0.001	56 (10%)	86 (28%)	79 (38%)	<0.001
Non-neurological complications, n (%)	170 (34%)	65 (39%)	16 (26%)	0.179	204 (28%)	263 (44%)	147 (44%)	<0.001	150 (27%)	139 (45%)	106 (51%)	<0.001
Pre-stroke anti-hyperglycemic therapy, n (%)	8 (2%)	45 (28%)	37 (62%)	<0.001	19 (3%)	37 (7%)	89 (30%)	<0.001	14 (3%)	27 (9%)	58 (29%)	<0.001
Post-stroke anti-hyperglycemic therapy, n (%)	29 (6%)	83 (49%)	58 (94%)	<0.001	40 (6%)	78 (13%)	151 (46%)	<0.001	34 (6%)	53 (17%)	118 (57%)	<0.001

HbA1c: hemoglobin A1c; RBG: random blood glucose; FBG: fasting blood glucose; BMI: body mass index; GCS: Glasgow Coma Scale; NIHSS: National Institutes of Health Stroke Scale.

Hematoma expansion was defined as an absolute increase in ICH volume greater than 6 ml or an increase of greater than 33% from baseline ICH volume.

The total number of patients in the row of hematoma expansion was denoted by N.

Surgical treatment included external ventricular drainage, microinvasive hematoma removal, craniotomy evacuation of hematoma and decompressive craniectomy.

Non-neurological complications included associated heart disease, infections, gastrointestinal bleeding and deep venous thrombosis.

There were significant statistical differences in GCS, NIHSS and hematoma volume among different RBG or FBG levels in univariate or multivariate logistic regression analysis (*P* < 0.001). Elevated RBG or FBG were associated with larger hematoma volume, lower GCS and higher NIHSS score. However, there was no significant difference of baseline GCS, NIHSS and hematoma volume in different HbA1c levels (*P* ˃ 0.05) in multivariate logistic regression analysis (Table 3).

**Table 3:** Association of glycemic status with hematoma volume, GCS and NIHSS after acute sICH.

Glycemic levels	Baseline hematoma volume				GCS				NIHSS			
	N	≥30 ml, n (%)	*P*_1_ value	*P*_2_ value*	N	≤12, n (%)	*P*_1_ value	*P*_2_ value*	N	≥16, n (%)	*P*_1_ value	*P*_2_ value*
HbA1c												
<6.0	403	97 (24%)	0.010	0.197	500	131 (26%)	0.750	0.579	500	98 (20%)	0.785	0.727
6.0–7.9	135	18 (13%)			168	44 (26%)			168	37 (22%)		
≥8.0	54	7 (13%)			62	19 (31%)			62	12 (19%)		
RBG												
<7.0	552	106 (19%)	<0.001	<0.001	720	216 (30%)	<0.001	<0.001	720	168 (23%)	<0.001	<0.001
7.0–9.9	401	147 (37%)			593	292 (49%)			593	247 (42%)		
≥10.0	199	77 (39%)			338	216 (64%)			336	197 (59%)		
FBG												
<6.0	488	89 (18%)	<0.001	<0.001	567	139 (25%)	<0.001	<0.001	567	102 (18%)	<0.001	<0.001
6.0–7.9	264	83 (31%)			307	122 (40%)			307	104 (34%)		
≥8.0	177	68 (38%)			208	125 (60%)			207	104 (50%)		

HbA1c: hemoglobin A1c; RBG: random blood glucose; FBG: fasting blood glucose; GCS: Glasgow Coma Scale; NIHSS: National Institutes of Health Stroke Scale.

*Adjusted for gender, age, history of hypertension, diabetes mellitus, history of dyslipidemia, anticoagulant or antiplatelet therapy, smoking, drinking, BMI, baseline systolic blood pressure and baseline hematoma location.

Of the 1805 patient with baseline data, there were 1515 patients with follow-up data, and other 290 patients were lost to follow up. There was no significant difference in baseline characteristics between the patients with or without follow-up information, except for age, prior ischemic stroke, prior use of antiplatelet agent and anti-hyperglycemic therapy (Table 1). Of the 1515 patients with available follow-up data, poor outcome of neurological function (mRS > 2) occured in 858 patients (56.6%), and 318 patients (21.0%) had died at 90 days after sICH. Logistic regression showed that poor outcome was associated with both elevated RBG and FBG (*P* < 0.001), but not associated with HbA1c (*P* = 0.616) in univariate analysis. Furthermore, the association between elevated levels of RBG and poor outcome was weaker than that of FBG, and did not persist in multivariable analysis when adjusted for gender, age, history of hypertension, diabetes mellitus, history of dyslipidemia, anticoagulant or antiplatelet therapy, smoking, drinking, BMI, baseline systolic blood pressure, GCS score, NIHSS score, baseline hematoma location and volume, intraventricular extension, surgical treatment, pre-stroke and post-stroke anti-hyperglycemic therapy (Table 4). Also, we provided data analysis after excluding the patients who died during the acute phase of ICH (within 14 days after ICH onset) as reference in supplementary material according to the reviewer’s request (Supplementary Table S1).

**Table 4:** Association of glycemic status with 90-day poor outcomes after acute sICH.

Glycemic levels	N	Events, n (%)	Univariate analysis		Multivariate analysis*	
			*P* value	OR	*P* value	aOR
HbA1c						
<6.0	437	193 (44.2)	0.616	1	0.850	1
6.0–7.9	150	67 (44.7)		1.02 (0.70–1.48)		0.79 (0.32–1.97)
≥8.0	56	21 (37.5)		0.76 (0.43–1.35)		0.69 (0.14–3.30)
RBG						
<7.0	591	266 (45.0)	<0.001	1	0.207	1
7.0–9.9	511	320 (62.6)		2.05 (1.61–2.61)		1.46 (0.87–2.46)
≥10.0	288	218 (75.7)		3.81 (2.78–5.21)		1.91 (0.83–4.40)
FBG						
<6.0	488	170 (34.8)	<0.001	1	0.021	1
6.0–7.9	280	167 (59.6)		2.76 (2.04–3.74)		2.22 (1.15–4.28)
≥8.0	174	123 (70.7)		4.51 (3.10–6.57)		3.20 (1.14–8.94)

HbA1c: hemoglobin A1c; RBG: random blood glucose; FBG: fasting blood glucose. Events: 90-day poor outcome.

*Adjusted for gender, age, history of hypertension, diabetes mellitus, history of dyslipidemia, anticoagulant or antiplatelet therapy, smoking, drinking, BMI, baseline systolic blood pressure, GCS score, NIHSS score, baseline hematoma location and volume, intraventricular extension, surgical treatment, pre-stroke and post-stroke hypoglycemic treatment.

Stratified analysis in patients with or without DM (including those diagnosed after the onset of ICH) showed that there were significant statistical differences in 90-day poor outcome among different FBG (*P* < 0.001) or RBG (*P* < 0.001) levels in univariate analysis, and among different FBG (*P* < 0.001) levels in multivariate analysis in patients without DM. However, in patients with DM, there was no significant statistical difference in 90-day poor outcome among different HbA1c, RBG or FBG levels in multivariate analysis (*P* > 0.05) (Table 5). In addition, compared with patients without DM, patients with DM had a higher proportion of poor outcome at 90 days after ICH onset, but the difference was not statistically significant (61.3% versus 55.6%, *P* = 0.077).

**Table 5:** Stratified analysis of association between glycemic status and 90-day poor outcomes after acute sICH in patients with or without DM.

Glycemic levels	Patients with DM						Patients without DM					
	N	Events (n, %)	Univariate analysis		Multivariate analysis*		N	Events (n, %)	Univariate analysis		Multivariate analysis*	
			*P* value	OR	*P* value	aOR			*P* value	OR	*P* value	aOR
HbA1c												
<6.0	16	11 (68.8)	0.131	1	>0.999	1	421	182 (43.2)	0.575	1	0.979	1
6.0–7.9	77	34 (44.2)		0.36		—	73	33 (45.2)		1.08		—
≥8.0	51	20 (39.2)		0.29		—	5	1 (20.0)		0.33		—
RBG												
<7.0	32	19 (59.4)	0.676	1	0.796	1	559	247 (44.2)	<0.001	1	0.108	1
7.0–9.9	68	42 (61.8)		1.11		0.45	443	278 (62.8)		2.13		1.38
≥10.0	157	104 (66.2)		1.34		0.97	131	114 (87.0)		8.47		3.26
FBG												
<6.0	26	9 (34.6)	0.026	1	>0.999	1	462	161 (34.8)	<0.001	1	0.015	1
6.0–7.9	51	24 (47.1)		1.68		—	229	143 (62.4)		3.11		2.34
≥8.0	100	62 (62.0)		3.08		—	74	61 (82.4)		8.78		4.34

HbA1c: hemoglobin A1c; RBG: random blood glucose; FBG: fasting blood glucose. Events: 90-day poor outcome.

*Adjusted for gender, age, history of hypertension, history of dyslipidemia, anticoagulant or antiplatelet therapy, smoking, drinking, BMI, baseline systolic blood pressure, GCS score, NIHSS score, baseline hematoma location and volume, intraventricular extension, surgical treatment, pre-stroke and post-stroke hypoglycemic treatment.

**Figure 1:**
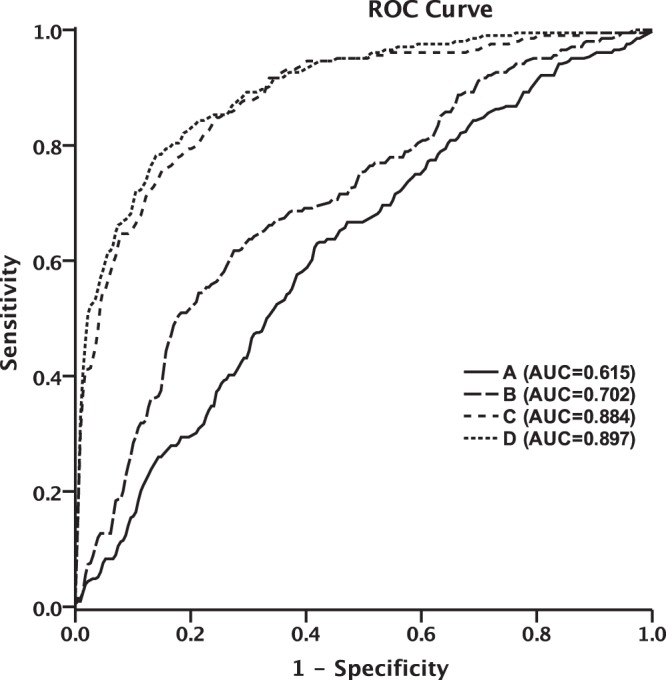
The ROC curve analysis of different glycemic levels for predicting poor clinical outcome. (**A**) ROC curve analysis of elevated RBG for predicting poor outcome (*P* < 0.001, AUC = 0.615); (**B**) ROC curve analysis of elevated FBG for predicting poor outcome (*P* < 0.001, AUC = 0.702); (**C**) ROC curve analysis of gender, age, history of hypertension, diabetes mellitus, history of dyslipidemia, anticoagulant or antiplatelet therapy, smoking, drinking, BMI, baseline systolic blood pressure, GCS score, NIHSS score, baseline hematoma location and volume, intraventricular extension, surgical treatment, pre-stroke and post-stroke hypoglycemic treatment, for predicting poor outcome (*P* < 0.001, AUC = 0.884). (**D**) ROC curve analysis of RBG and FBG, combined with gender, age, history of hypertension, diabetes mellitus, history of dyslipidemia, anticoagulant or antiplatelet therapy, smoking, drinking, BMI, baseline systolic blood pressure, GCS score, NIHSS score, baseline hematoma location and volume, intraventricular extension, surgical treatment, pre-stroke and post-stroke anti-hyperglycemic therapy, for predicting poor outcome (*P* < 0.001, AUC = 0.897).

The ROC curve analysis of elevated FBG and RBG for predicting poor outcome showed that FBG was superior to RBG, and the area under the curve (AUC) was 0.702 (*P* < 0.001) and 0.615 (*P* < 0.001) respectively. Furthermore, the ROC curve analysis of elevated RBG and FBG, combined with gender, age, history of hypertension, diabetes mellitus, history of dyslipidemia, anticoagulant or antiplatelet therapy, smoking, drinking, BMI, baseline systolic blood pressure, GCS score, NIHSS score, baseline hematoma location and volume, intraventricular extension, surgical treatment, pre-stroke and post-stroke anti-hyperglycemic therapy, for predicting poor outcome showed that the AUC was 0.897 (*P* < 0.001) (Fig. 1).

**Figure 2:**
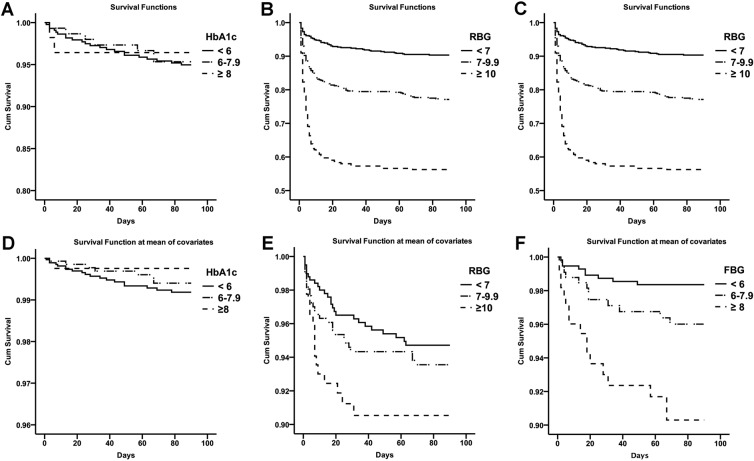
Kaplan-Meier curve and Cox regression analysis of different glycemic levels for survival. (**A**–**C**) Kaplan-Meier curve (log-rank test) demonstrated that there were significant differences among three survival curves of FBG (*P* < 0.001, HR (fair/perfect) = 3.01, HR (poor/perfect) = 6.81) and RBG (*P* < 0.001, HR (fair/perfect) = 2.56, HR (poor/perfect) = 5.66), but no significant difference among three survival curves of HbA1c (*P* = 0.893); (**D**–**F**). Cox regression analysis demonstrated that there were still significant differences among three survival curves of FBG (*P* = 0.042) and RBG (*P* < 0.001) when adjusted for gender, age, history of hypertension, diabetes mellitus, history of dyslipidemia, anticoagulant or antiplatelet therapy, smoking, drinking, BMI, baseline systolic blood pressure, GCS score, NIHSS score, baseline hematoma location and volume, intraventricular extension, surgical treatment, pre-stroke and post-stroke anti-hyperglycemic therapy.

Kaplan-Meier curve for survival (log-rank test) demonstrated that there were significant differences among three survival curves of FBG (*P* < 0.001, HR (fair/perfect) = 3.01, HR (poor/perfect) = 6.81) and RBG (*P* < 0.001, HR (fair/perfect) = 2.56, HR (poor/perfect) = 5.66), but no significant difference among three survival curves of HbA1c (*P* = 0.893). Compared with perfect FBG (<6.0 mmol/L), fair (6.0–7.9 mmol/L) or poor FBG (FBG ≥8.0 mmol/L) increased the risk of death within 90 days after sICH onset. In addition, compared with perfect RBG (<7.0 mmol/L), fair (7.0–9.9 mmol/L) or poor RBG (FBG ≥10.0 mmol/L) also increased the risk of death within 90 days (Fig. 2).

Cox regression analysis demonstrated that there were still significant differences among three survival curves of FBG (*P* = 0.042) and RBG (*P* < 0.001) when adjusted for gender, age, history of hypertension, diabetes mellitus, history of dyslipidemia, anticoagulant or antiplatelet therapy, smoking, drinking, BMI, baseline systolic blood pressure, GCS score, NIHSS score, baseline hematoma location and volume, intraventricular extension, surgical treatment, pre-stroke and post-stroke anti-hyperglycemic therapy (Fig. 2).

## Discussions

In this multicenter, prospective, observational cohort study, we disclosed the association of pre- and post-stroke glycemic status with clinical outcome in patients with acute sICH. We found that elevated FBG or admission RBG were associated with larger hematoma volume, more severe neurological damage at admission, and 90-day poor outcome. Furthermore, the association of 90-day poor outcome with elevated RBG or FBG retained statistical significance in the patients without DM, but not in the patients with DM. In contrast, elevated prestroke glycemic levels, represented by HbA1c, was associated with neither hematoma volume, neurological status at admission, nor 90-day poor outcome in patients with acute sICH.

Poststroke hyperglycemia has been thought to be related to hematoma expansion, presence and severity of intraventricular extension, perihematomal edema and poor clinical outcome in patients with acute sICH^3–5,7,16,17^. However, some studies indicated that high admission glucose level signified the severity of stroke but it was not a predictive factor for prognosis of stroke^12,18^, and early intensive insulin therapy had failed to improve functional outcome after stroke in previous studies^8,9^. In this study, elevated RBG or FBG was associated with larger hematoma volume, higher GCS and NIHSS score. The 90-day poor outcome was associated with both elevated RBG or FBG (*P* < 0.001). In stratified analysis, the association of 90-day poor outcome with elevated RBG or FBG retained statistical significance in the patients without DM, but not in the patients with DM, which was similar with the results of previous studies^1,19^. Results from the INTERACT2 study suggested that hyperglycemia and DM are independent predictors of poor outcome in patients with ICH^7^. However, our results showed that patients with DM had a higher proportion of poor outcome at 90 days after ICH onset, but the difference was not statistically significant (61.3% versus 55.6%, *P* = 0.077). ROC curve analysis demonstrated that RBG and FBG, combined with gender, age, history of hypertension, diabetes mellitus, history of dyslipidemia, anticoagulant or antiplatelet therapy, smoking, drinking, BMI, baseline systolic blood pressure, GCS score, NIHSS score, baseline hematoma location and volume, intraventricular extension, surgical treatment, pre-stroke and post-stroke hypoglycemic treatment, can better predict the risk of poor outcome in patients with acute sICH (*P* < 0.001, AUC = 0.897).

Furthermore, the association between high levels of RBG and poor outcome was weaker than that of FBG, and did not persist after multivariable assessment. The ROC curve analysis also showed that FBG was superior to RBG for predicting poor clinical outcome, and this phenomenon was also indicated in patients with aneurysmal subarachnoid hemorrhage^20,21^. This may be explained that RBG might not accurately reflect an individual’s long-term status, owing to its limitation of great intraindividual variation.

The exact pathophysiological mechanism underlying the relation between poststroke hyperglycemia and severe neurological deficit or poor outcome in patients with sICH is still unclear. It is really indeterminate whether higher glucose is just a consequence of severe neurologic damage, or actually independently contributes to it. Some studies suggested that the poststroke hyperglycemia may result from stress reaction, especially adrenergic stress and relative insulin deficiency, suggesting that poststroke hyperglycemia could only reflect severity of the ICH as a stress reaction to a serious brain injury, and may be just a surrogate marker for severe hemorrhagic stroke^22–24^. Some studies, including the present study revealed that the association of poor outcome with elevated RBG or FBG was significant in the patients without DM, but not in the patients with DM, which could support the explanation of stress reaction to some extent^1,19^. In patients without DM, post-stroke hyperglycemia in the acute phase of ICH may be more attributed to stress response, while in patients with DM, hyperglycemia may be related to diabetes itself. At the same time, some studies do suggest that poststroke hyperglycemia directly result in neurological injury^25,26^. It is reported that acute hyperglycemia increases circulating cytokine concentrations by an oxidative mechanism^27^. Another study showed that in ICH patients after hematoma evacuation, intensive blood glucose control was associated with lower extracellular lactate glutamate and pyruvate levels, which may be related to early hematoma expansion and poor outcomes in patients with ICH^28,29^. It is also reported that in experimental ICH animals, hyperglycemia itself could contribute to neurological damage, causing downregulated Aquaporin-4 (AQP-4) expression, abnormal calcium metabolism, more severe blood-brain barrier destruction, profound vasogenic brain edema and perihematomal cell death^25,26,30^.

Based on the results of this study, whether higher glucose is just a consequence of severe neurologic damage, or actually contributes to it, current guideline recommendations for good glycemic control in patients with intracerebral hemorrhage is still the good choice^10^. However, the optimal management of hyperglycemia in ICH and the target glucose level still remains to be clarified.

There is no denying the fact that many factors influence blood glucose levels, so the blood glucose levels fluctuate constantly over time. In addition, the interval from ICH onset to measurement of blood glucose value might vary from one patient to another. So it is difficult to standardize the blood glucose levels, and we can only compare it at a relatively specific period of time. In contrast, as a biomarker of prestroke long-term (about 3 months) glycemic status, HbA1c is relatively stable than acute blood glucose and widely used for monitoring diabetic vascular damage, including atherosclerosis and microangiopathic changes, and HbA1c was reported to be independently associated with poor outcome of ischemic stroke^31^. It was suggested that chronic exposure, rather than short-term exposure, to hyperglycemia could contribute to vascular damage and unfavorable effects on sICH^32^. Few studies conducted in small cohorts had suggested that HbA1c could serve as a better predictor of large hematoma volume and poor outcome in patients after sICH^5,12,13^, and may be a better predictor of symptomatic hemorrhagic transformation after thrombolysis for acute ischemic stroke^13^. A large community-based cohort study revealed that low (<4.0 mmol/L) and high (≥6.1 mmol/L) fasting blood glucose concentrations were associated with higher risk of incident ICH, which suggests to some extent that prestroke blood sugar levels may affect the ICH morbidity and prognosis^32^. In the present study, however, there was no significant difference of baseline GCS, NIHSS, hematoma volume, 90-day poor outcome or death in different HbA1c levels (*P* ˃ 0.05). But we noticed that there were significant differences in GCS, NIHSS, hematoma volume, proportion of IVH extension and mortality rate between the valid and missing groups of HbA1c. The patients in missing group had more severe neurological deficits, larger hematoma volume and worse outcome. So we cannot rule out the possibility that the risk effect of HbA1c may be underestimated.

Potential limitations of our study should be mentioned. First, a significant difference was observed in the hematoma volume and mortality rate between the patients with or without HbA1c measurement. However, the hematoma volume had been adjusted in the multivariable analysis. Second, blood glucose levels were evaluated over a specific period of time, rather than a specific point of time in consideration of intraindividual variation of glycemic status. So it necessitates further prospective cohorts or randomized studies to confirm our conclusions.

## Conclusions

In this study, we found that poststroke hyperglycemia was associated with larger hematoma volume, severe neurological damage and poor clinical outcome, but prestroke hyperglycemia (reflected by HbA1c) was not relevant to hematoma volume, neurological damage or clinical outcome in patients with sICH.

## Supplementary information

Supplementary material

## Data Availability

The data that support the findings of this study are available from the corresponding author upon reasonable request.
