# Supplementary material for: Association of pre- and post-stroke glycemic status with clinical outcome in spontaneous intracerebral hemorrhage
Source: Sci Rep. 2019 Dec 13;9:19054. doi: 10.1038/s41598-019-55610-z (PMC6911072; doi:10.1038/s41598-019-55610-z)

Supplementary material

Association of pre- and post-stroke glycemic status with clinical outcome in spontaneous intracerebral hemorrhage

Kaijiang Kang, MD[1]; Jingjing Lu, MD[1]; Yi Ju, MD[1]; Wenjuan Wang, MD[1]; Yuan Shen, MD[1]; Anxin Wang, MD[2]; Zhentang Cao, MD[1];

Xingquan Zhao, MD[1]

[1] Department of Neurology, Beijing Tiantan Hospital, Capital Medical University, China National Clinical Research Center for Neurological Diseases, Center of Stroke, Beijing Institute for Brain Disorders, Beijing, China.

[2] China National Clinical Research Center for Neurological Diseases, Beijing, China.

Corresponding authors: Prof. Xingquan Zhao, Department of Neurology, Beijing Tiantan Hospital, Capital Medical University, No.6 Tiantanxili, Dongcheng District, Beijing, China, 100050 (tel: 86-10-59975701; fax: 86-10-67013383; E-mail: zxq@vip.163.com). ORCID：0000-0002-8636-9540

Number of supplementary tables: 1

Number of supplementary figures: 1

Supplementary Table S1. Association of glycemic status with 90-day poor outcomes after exclusion of patients who died within 14 days after sICH onset.

Glycemic levels		N	Events, n (%)	Univariate analysis		Multivariate analysis*	
				P value	OR	*P* value	aOR
HbA1c	＜6.0	429	185 (43.1)	0.507	1	0.863	1
	6.0-7.9	148	65 (43.9)		1.03 (0.71-1.51)		0.80 (0.32-2.00)
	≥8.0	54	19 (35.2)		0.72 (0.40-1.29)		0.70 (0.15-3.35)
RBG	＜7.0	555	230 (41.4)	＜0.001	1	0.573	1
	7.0-9.9	423	232 (54.8)		1.72 (1.33-2.22)		1.31 (0.76-2.26)
	≥10.0	174	104 (59.8)		2.10 (1.48-2.97)		1.39 (0.57-3.39)
FBG	＜6.0	479	161 (33.6)	＜0.001	1	0.083	1
	6.0-7.9	263	150 (57.0)		2.62 (1.93-3.57)		2.04 (1.04-3.99)
	≥8.0	146	95 (65.1)		3.68 (2.49-5.43)		2.29 (0.78-6.72)

HbA1c: hemoglobin A1c; RBG: random blood glucose; FBG: fasting blood glucose. Events: 90-day poor outcome.

*Adjusted for gender, age, history of hypertension, diabetes mellitus, history of dyslipidemia, anticoagulant or antiplatelet therapy, smoking, drinking, BMI, baseline systolic blood pressure, GCS score, NIHSS score, baseline hematoma location and volume, intraventricular extension, surgical treatment, pre-stroke and post-stroke hypoglycemic treatment.

Supplementary Figure S1. Overview of the included patients in the study and in each subset of analysis

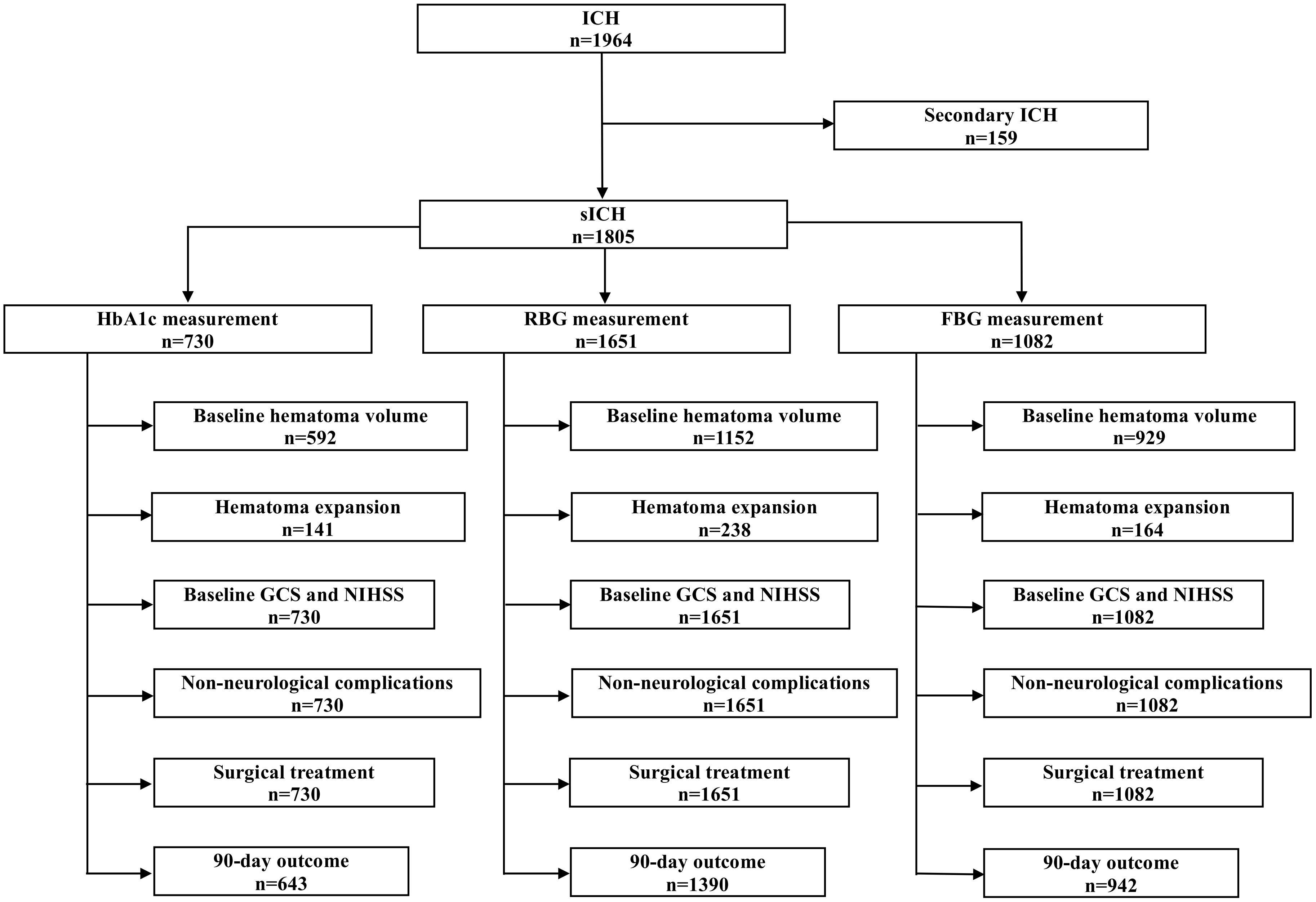

Supplement: Supplementary file 1 — Supplementary material [file 41598_2019_55610_MOESM1_ESM.pdf]
